# Evaluation of rotavirus vaccine administration among a 22q11.2DS patient population

**DOI:** 10.1186/s13223-022-00693-z

**Published:** 2022-06-11

**Authors:** Sophie McGregor, Matthew Boroditsky, Geraldine Blanchard-Rohner, Christine Loock, Kyla Jade Hildebrand

**Affiliations:** 1 Medical Undergraduate Program, University of British Columbia, Kelowna, BC Canada; 2 Division of Plastic Surgery, University of British Columbia, Vancouver, BC Canada; 3 Unit of Immunology and Vaccinology, Division of General Pediatrics, Department of Pediatrics, Gynecology and Obstetrics, Geneva University Hospitals, University of Geneva, Geneva, Switzerland; 4 Department of Pediatrics, Faculty of Medicine, University of British Columbia, Vancouver, BC Canada; 5 British Columbia Children’s Hospital Research Institute, 4480 Oak St, Vancouver, BC Canada; 6 British Columbia Children’s Hospital, Division of Allergy and Immunology, Department of Pediatrics, Faculty of Medicine, University of British Columbia, Vancouver, BC Canada

**Keywords:** 22q11.2DS, Vaccines, Rotavirus vaccination

## Abstract

**Background:**

22q11.2 Deletion Syndrome (22q11.2DS) can result in array of congenital abnormalities including immune dysfunction. International guidelines recommend immune evaluation of 22q11.2DS patients prior to live vaccine administration. A rotavirus vaccination program for infants aged 2 and 4 months was implemented in British Columbia (BC) in 2012. Adherence to immune workup recommendations prior to 2 months of age in patients with 22q11.2DS and adverse events following immunization is not known.

**Methods:**

A retrospective chart review of children diagnosed with 22q11.2DS in BC from January 1, 2012 to January 1, 2019 was conducted. Demographic, clinical, laboratory, immunization data and adverse reactions to vaccines were obtained. International guidelines were used as a reference for adherence to immunologic workup recommendations.

**Results:**

Forty-two children with 22q11.2DS were included. Immunization records were available for 39 children, and 22 (52.3%) received at least one dose of a live rotavirus vaccine. No adverse events following immunization were noted in clinical records. While 25 out of 27 (92.6%) of patients who received an immunological workup had a CD4 + lymphocyte count to qualify for safe administration of a live vaccination, only 12 (44%) received the Rotavirus vaccine. Of 22 infants diagnosed with 22q11.DS prior to 8 weeks of age, only ten (45.5%) received an immune workup before the rotavirus vaccine.

**Conclusions:**

The majority of our infant cohort did not receive medical care consistent with international 22q11.2DS vaccination and immunological surveillance recommendations. More effective dissemination of 22q11.2DS guidelines and improved immunological assessment for infants with 22q11.2DS in BC is necessary.

## Background

22q11.2 Deletion Syndrome (22q11.2DS), previously referred to as DiGeorge’s Syndrome, has a prevalence of roughly 1:1000–1:2000 live births [1]. Individuals with 22q11.2DS have an array of congenital anomalies and health conditions, including features of cardiovascular, craniofacial anomalies; hypoparathyroidism; skeletal and renal anomalies; and neurodevelopmental delays [2, 3]. Also, most children with 22q11.2DS have a varying degree of diminished T-cell numbers and thymic hypoplasia with mild to moderate immune dysregulation [1–3]. Immunodeficiency within the 22q11.2DS patient population remains a cornerstone of early patient evaluation and management, affecting up to 75% of the patients with the syndrome [2]. Approximately 0.5% of 22q11.2DS patients present with severe immunodeficiency associated with complete thymic aplasia [4].

Children with 22q11.2DS are at increased risk of infections from vaccine-preventable diseases, thus routine childhood immunizations are an important preventative health measure [5]. Among severely immunocompromised people, live vaccines have the potential to cause infection in the host [6]. However, studies have shown the efficacy and safety of live vaccines such as Measles, Mumps, and Rubella (MMR) and Varicella in 22q11.2DS patients with a CD4 + count greater than 500 × 10^6^ cells/L [5, 7–9]. The National Advisory Committee on Immunization has published the Canadian Immunization Guide and recommends that individuals with 22q11.2DS who have a total lymphocyte count of  > 500 × 10^6^ cells/L may receive a live MMR vaccine [10], while the Infectious Diseases Society of America (IDSA) guideline recommends that live vaccines can be safely administered in patients with 22q11DS if the total T cell (CD3 +) count is more than 500 × 10^6^ cells /L, helper T cell (CD4 +) is more than 500 × 10^6^/L, cytotoxic T cell (CD8 +) count is more than 200 × 10^6 ^cells/L, and a normal mitogen response has been demonstrated.

The rotavirus vaccination program was implemented as part of British Columbia’s (BC) immunization schedule in January 2012 [11]. The first dose of the rotavirus vaccine is scheduled at 2 months of age, or as early as 6 weeks [12]. This differs from other scheduled live vaccines in BC, which are administered 12 and 18 months [13]. There is a paucity of information in the literature surrounding the safety of the rotavirus vaccine in infants with 22q11.2DS. Currently, the rotavirus vaccine is contraindicated in children with partial or full T-cell deficiencies until their immune competence has been established [10].

International 2011 22q11.2DS guidelines recommend immunological assessments for patients with 22q11.2DS including flow cytometry in newborns and then annual complete blood count (CBC) with differential, flow cytometry, immunoglobulin count, and T-cell function assay at diagnosis and/or prior to live vaccine administration [3]. Nevertheless, there is heterogeneity in the extent of immunologic workup [14]. Traditionally, infants with 22q11.DS should have received an immune workup prior to 12 months of age when the MMR vaccine is due to be administered [3]. However, the early age of administration of the rotavirus vaccine creates a challenge in meeting these requirements, as children with milder phenotypes of 22q11.2DS may not be diagnosed until after live vaccine administration [15, 16]. Additionally, whether care providers are obtaining immune workups for their patients with known 22q11.2DS prior to rotavirus administration at months 2 of age is not known.

The objectives of our study were threefold: First, to assess how many patients within our cohort received a rotavirus vaccination. Second, to assess whether 22q11.2DS infants who were vaccinated against rotavirus presented with any vaccine adverse events following immunization. Third, evaluate the adherence to and timing of the immunological workup, as published in the 2011 pediatric 22q11.2DS guidelines [3]. We hypothesized that patients with a known diagnosis of 22q11.2DS at the time of rotavirus vaccination would be less likely to receive the vaccine compared to those without a diagnosis, those who received the rotavirus vaccine would have minimal to no adverse events, and that immunologic workup is not a routine practice prior to live vaccine administration in the 22q11.DS population.

## Methods

The study protocol was approved by the University of British Columbia’s Women’s and Children’s Research Ethics Board (H20-03,309). We included patients with 22q11.2DS born from January 1, 2012 to January 1, 2019, in alignment with the rotavirus rollout in BC, who received care through BCCH or BC Women’s Hospital. This BCCH database was previously established from an alternative 22q11.2DS study, analysing the subspecialty services and care requirements for these patients [16]. Patients were identified by searching the discharge database for patients diagnosed with “DiGeorge syndrome” or “22Q deletion/transition” (ICD-10 code D82.1). Patients were also queried from Population Data BC for “velocardiofacial syndrome” (ICD-9 code 758.32) and “DiGeorge syndrome” (ICD-9 code 279.11 and ICD-10 code D82.1). Distinct specialty service databases caring for patients with 22q11.2DS at BCCH were also included. For a more detailed outline of this methodology, refer to [16].

### Outcome measures

Individual charts were manually reviewed to obtain demographic, clinical, laboratory, and immunization data.

Age at diagnosis was defined as age in which first mention of 22q11.2DS diagnosis was made in charts, or if available, the date of a positive cytogenetics report. Since maternal patient information was not available, patients with a prenatal diagnosis of 22q11.2DS were categorized as having a diagnosis at 0 weeks old.

Immunization data was collected from patient eHealth database. Adverse events to immunizations were defined as either a documented Adverse Event Following Immunization (AEFI) in patient’s provincial e-database records (CareConnect eHealth viewer) of AEFI prompting escalation of care to a specialist or the emergency department.

International consensus guidelines were used as a reference point to follow adherence to guidelines for immunologic workup. We obtained patients’ first documented flow cytometry and CBC with differential results. Additionally, white blood cell, lymphocyte, CD3  + , CD4  + , CD8  + , and CD19  + cell counts were recorded.

## Results

**Fig. 1:**
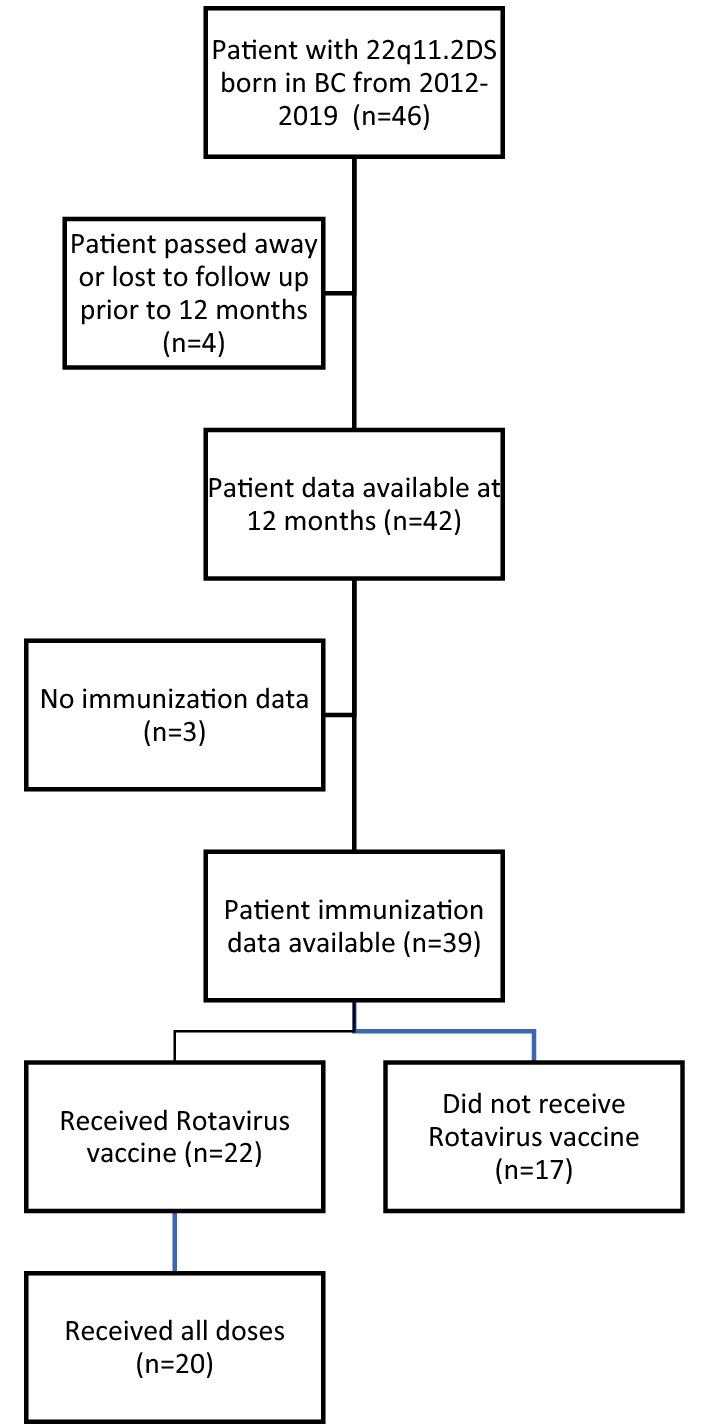
Flow chart of study population selection

We identified 46 patients with 22q11.2DS between January 1, 2012, and January 1, 2019. Four charts were excluded as one patient was lost to follow-up and three patients passed away prior to one year of age (Fig. 1). Twenty-six (56.5%) were female. Nineteen (41%) patients were diagnosed with 22q11.2DS at or prior to birth, and the median age of diagnosis was 5 weeks (IQR 0, 76 n = 42). Twenty-three (54.8%) patients were diagnosed with 22q11.2DS before they were 8 weeks old. Immunization records were available for 39 (92.9%) of the patients. Twenty (56.4%) children had surgery within the 1st year of life related to 22q11.2DS complications (18 cardiac surgeries, 1 otolaryngologic surgery, and 1 gastroenterological surgery).

### Immunological assessment

**Fig. 2:**
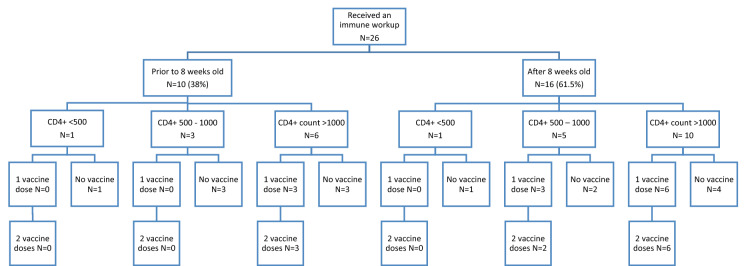
Flow chart of rotavirus vaccination depending on initial documented CD4  + (× 10^6^ cells/L) count

Twenty-six (66.7%) patients had a CBC and differential and flow cytometry performed at least once, and ten (23.8%) patients had these investigations prior to 8 weeks of age, before the scheduled time of rotavirus vaccine administration, though 7 (16.7%) did not receive a rotavirus vaccine (Fig. 2). Most patients had mild, or no immunodeficiency as indicated by their total lymphocyte and CD4  + counts.

Of the 26 who received an immune workup, two (7.7%) patients had a CD4  + count below 500 × 10^6^/L (400 × 10^6^ cells/L and 420 × 10^6^ cells/L), neither of whom received a rotavirus vaccine. Two infants had a CD8 + count lower than 200 × 10^6^/L (116 × 10^6^ cells/L and 110 × 10^6^ cells/L), one of whom received two doses of the rotavirus vaccine. Figure 2 depicts the course of patients’ rotavirus vaccination status based on their CD4 + count. No patients had a CD3 + lymphocyte count less than 500 × 10^6^ cells/L.

Of 20 children who had surgery in the 1st year of life, 90% (18/20) received an immune workup, while only 42% (8/19) of patients who did not undergo surgery had immunological testing. However, whether or not they had surgery did not influence their likelihood to receive the rotavirus vaccine regardless of their reported immune status (11/20 and 11/19 received a rotavirus vaccine, respectively).

### Immunization status

Of the 39 patients whose immunization records were available, 22 (56.4%) received at least one dose of a live rotavirus vaccine (Table 1). Of 17 patients who received no doses of the rotavirus vaccine, 8 (47.0%) received at least one live vaccine at 12 months. Out of 19 infants who were diagnosed with 22q11.2DS prior to 8 weeks, 47.4% (n = 9) received the first dose of the rotavirus vaccine, 8 (88.9%) of whom completed their rotavirus vaccine schedule at 4 months. In comparison, 65.0% (n = 13) of those who were diagnosed with 22q11.2DS after 8 weeks of age received a rotavirus vaccine.

**Table 1:** Vaccine schedule adherence among infants with 22q11.2DS

	Population (N = 39)	Percent
Vaccine schedule adherence^a^		
2 months^b^	21	53.8%
4 months^c^	21	53.8%
6 months^d^	28	71.7%
12 months^e^	28	71.7%
Rotavirus vaccine received	22	56.4%
2 Rotavirus doses received	20	51.2%
Median age of first dose (weeks)	8	IQR (8,9)
Patients who received live vaccines	30	76.9%

^a^Vaccine schedule adherence define as whether patient received all vaccines recommended by the immunize BC guidelines [13] for each specified age

^b^Received all recommended vaccines at 2 months: DTaP-HB-IPV-HiB, Pneumococcal conjugate, Rotavirus, Meningococcal C conjugate

^c^Received all recommended vaccines at 4 months: DTaP-HB-IPV-HiB, Pneumococcal conjugate, Rotavirus

^d^Received all recommended vaccines at 6 months: DTaP-HB-IPV-HiB

^e^Received all recommended vaccines at 12 months: Pneumococcal conjugate, Meningococcal C conjugate, MMR, and Varicella

### Adverse reactions

There were no reported adverse reactions in our cohort, as documented in AEFI reports, specialist consultation, or ED visits in patient EMRs.

### Adherence to international 22q11.DS guidelines

When analysing the entire cohort (n = 39), 38.5% received an immune function workup prior to any live vaccine administration, and 46.2% received an immune workup at diagnosis of 22q11.2DS. Twenty-seven (69.2%) patients received a CBC with differential at the time of diagnosis.

Of the 19 patients diagnosed with 22q11.2DS before 8 weeks of age, only ten (52.6%) infants received an immune workup before the age of first Rotavirus vaccine.

## Discussion

With the introduction of rotavirus vaccinations to the BC immunization schedule in 2012, we sought to determine the proportion of patients with 22q11.2DS who received a rotavirus vaccine and if any of them experienced adverse reactions*,* and we evaluated adherence to the 22q11.2DS immunological workup guidelines [2, 3]. More than half (56.4%) of our population received a rotavirus vaccination, and there were no documented adverse events in patient charts. None of our patients presented with severe immune compromise or a total lymphocyte count lower than 500 × 10^6^ cells/L, and only two patients had CD4  + counts below 500 × 10^6^ cells/L.

Our study highlights two key findings: first, 80% of children who received an immunological assessment prior to rotavirus vaccination had a sufficient CD4  + lymphocyte count to qualify for safe administration of live vaccines; however, most did not receive the rotavirus vaccine. Second, 47.4% of infants with a known 22q11.2DS diagnosis (n = 19) did not receive immunological assessment prior to live vaccine administration.

Children with a mild to moderate immune deficiency often have increased frequency of persistent infections, but the risk of live vaccines is shown to be low except in those with complete thymic aplasia [5, 7–9]. Currently, there are no studies of rotavirus vaccine safety in infants with 22q11.2DS, but prospective trials have demonstrated sufficient safety of rotavirus vaccine in infants with HIV with CD4 + lymphocyte counts higher than the 500 × 10^6^ cells/L [17, 18]. In our population, 42.3% of patients with CD4 + counts above 500 × 10^6^ cells/L did not receive a rotavirus vaccine despite meeting the IDSA immune count criteria. In BC, rotavirus vaccination coverage in the general population ranges from 73 to 80% of infants per year [19]. It is possible that the physicians were unaware of the lymphocyte count cut-offs for immunization when they ordered flow cytometry and were cautious in their vaccine recommendations. Parental vaccine hesitancy also influences vaccine uptake [20], therefore the benefits of the rotavirus vaccine in infants with 22q11.2DS and normal immune system should be emphasized to physicians, allied providers, and caregivers alike.

While there were no reported adverse effects to the rotavirus vaccination in our population, an immune workup prior to live vaccination is imperative for early identification of severe T cell compromise in 22q11.2DS patients. This would ideally mitigate side effects, such as severe diarrhea, reported in infants with severe immunocompromise who received a rotavirus vaccine [21]. Canadian provinces except BC, Saskatchewan, and Quebec, have implemented newborn screening (NBS) for Severe Combined Immunodeficiency (SCID) by using T-cell receptor excision circle (TREC) assays, which measures T cells receptor creation. Infants with profound lymphocyte dysfunction have inadequate numbers of TRECs, and while TREC screening would not catch all 22q11.2DS patients who have lymphocyte counts below the IDSA cut-off, it would provide early identification of severe cases of thymic aplasia secondary to 22q11.2DS [22].

Nevertheless, the major challenge remains that most 22q11.2DS diagnoses continue to be made after 8 weeks of age [16]. Chromosomal microarray is the gold standard for diagnosis of 22q11.2DS and should be offered to all infants with multiple congenital anomalies as early as possible [23].

Patients with 22q11.2DS have complex care needs and are seen on average by five to seven specialists [15, 16]. The international 22q11.2DS guidelines were created as a resource for screening, evaluation, and management of these children [3]. The implementation of the infant rotavirus vaccine schedules poses a challenge to immunologic spheres of these guidelines. We found that adherence to the immunological guidelines was low. The complexity of care required, with limited integrated case management may be driving poor adherence to guidelines [20], further emphasizing the role for a more intersectional, holistic approach [24]. We did note that patients who had complications from 22q11.2DS requiring surgical intervention in the 1st year of life were more likely to have an immune workup performed. It is possible that the need for surgery and pediatric intensive immunological care correlates for more frequent medical visits and investigations, and by proxy they may be more connected with BCCH services.

## Limitations

Our study has several limitations. The observational nature of our cohort, with patients seen across multiple providers and sites, limits the consistency and standardization in reporting [16]. Additionally, our passive surveillance of AEFI’s is limited by potential underreporting of adverse reactions. Our entire vaccinated patient cohort received the Rotarix vaccine (GlaxoSmithKline Biologicals, Belgium), a monovalent vaccine with scheduled doses at 2 and 4 months. In April of 2018, BC began to use the pentavalent Rotateq vaccine (Merck Canada, Kirkland, Canada), which recommends three doses [11], and while the safety profile is reported to be comparable [25], there may be differences in tolerance between either vaccine. Also, we did not collect data on the disposition of infants at the time of their scheduled rotavirus vaccine. It is possible that infants who were hospitalized for conditions relating to their medical comorbidities at 2 or 4 months of age did not receive the rotavirus vaccine due to the risk of vaccine virus shedding [10].

Finally, although the international 22q11.2DS and IDSA guidelines recommend T-cell function tests as part of the immune workup prior to live vaccines [3, 6], we only investigated the flow cytometry adherence, as T-cell function testing is not a routine practice in BC due to it is limitations in access. Quantitative T-cell measurement alone may be enough to determine eligibility of live viral vaccine administration, particularly when NBS for TRECs is universally available in Canada, and revision of practice guidelines for this patient population may be warranted.

## Conclusions

Standardized care for 22q11.2 DS is evolving. We found that most of our cohort had not received care in line with the international 22q11.2DS guidelines. Most infants who received an immunological workup prior to their rotavirus vaccine did not go on to receive the rotavirus vaccine despite having sufficient CD4 + lymphocyte counts. While almost half (47.4%) of the infants with known 22q11.2DS had no immunological workup, they were administered a rotavirus vaccine; no adverse reactions were reported in any patients who received a rotavirus vaccine. Further assessment of guidelines to determine whether immunological workup prior to rotavirus vaccine is necessary for infants with 22Q11.2 DS in locations where TREC assays are performed for all infants at birth is warranted. Additionally, more effective dissemination of 22q11.2DS guidelines and improved infant screening for 22q11.2DS in BC is necessary. Provincial 22q11.2DS centres of excellence would ensure more integrated care for these patients and would likely improve adherence to evidence-based guidelines.

## Data Availability

The datasets analysed during this study are available upon reasonable request.

## Abbreviations

22q11.2DS: 22Q11.2 Deletion syndrome
BC: British Columbia
